# Supplementary material for: Association between oral microbiome and seven types of cancers in East Asian population: a two-sample Mendelian randomization analysis
Source: Front Mol Biosci. 2023 Nov 21;10:1327893. doi: 10.3389/fmolb.2023.1327893 (PMC10702768; doi:10.3389/fmolb.2023.1327893)
Supplement: Supplementary file 1 [file DataSheet1.ZIP › Supplementary Materials/MR plots for tongue/tongue═╝/Gastric cancer/pheno.1299_to_gastric cancer_forest.pdf]

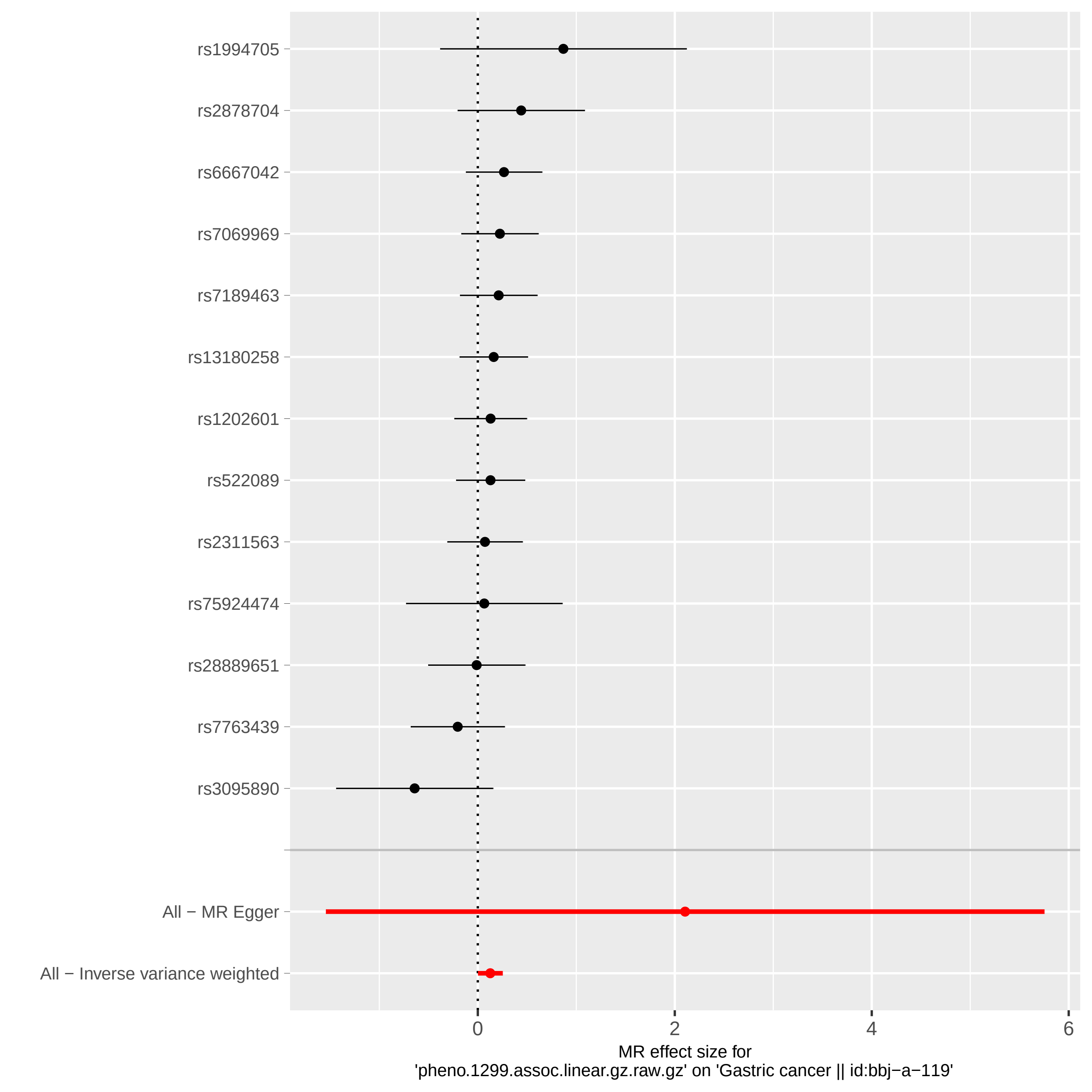

rs1994705
rs2878704
rs6667042
rs7069969
rs7189463
rs13180258
rs1202601
rs522089
rs2311563
rs75924474
rs28889651
rs7763439
rs3095890
All − MR Egger
All − Inverse variance weighted
0
2
4
6
MR effect size for
'pheno.1299.assoc.linear.gz.raw.gz' on 'Gastric cancer || id:bbj−a−119'
